# Supplementary material for: Development and validation of new evaluation scale for measuring stroke patients’ motivation for rehabilitation in rehabilitation wards
Source: PLoS One. 2022 Mar 17;17(3):e0265214. doi: 10.1371/journal.pone.0265214 (PMC8929594; doi:10.1371/journal.pone.0265214)
Supplement: S1 Appendix — Details of first and second round Delphi method results. Item 10 and 14 were excluded from MORE scale because less than 80% of the participants’ consensus. (DOCX) [file pone.0265214.s001.docx]

**S1 Appendix. Percentage of item consensus by Delphi method.**

| Item |  | First round (%) | Second round (%) |
| --- | --- | --- | --- |
| 1 | I want to participate in rehabilitation for my goals. | 96.4 | 97.5 |
| 2 | I do not want to discharge until I achieve my recovery objectives. | 85.5 | 85.0 |
| 3 | I want to do train in order to regain my role in my home and our society. | 100.0 | 100.0 |
| 4 | I am able to make efforts to achieve my goal. | 80.7 | 82.5 |
| 5 | I want to work hard to meet my therapists’ expectations. | 74.7 | 80.0 |
| 6 | I want to use of the abilities I regained from the rehabilitation process in my daily life. | 95.2 | 96.3 |
| 7 | I share my daily exercise target with my therapist on a daily basis. | 89.2 | 88.8 |
| 8 | Alternations of daily rehabilitation plans propel me to participate more. | 92.8 | 93.8 |
| 9 | I was encouraged by other patients’ effort. | 97.6 | 97.5 |
| 10 | I think rehabilitation does not improve my body function, but rather makes it worse. (reversed item) | 55.4 | 58.8 |
| 11 | I want to participate in rehabilitation for the sake of my friends and family. | 96.4 | 97.5 |
| 12 | I feel my body functions (such as body movement) improve on a daily basis. | 92.8 | 95.0 |
| 13 | I would like to keep practicing so that I can regain my ability to perform lost/unexecuted daily activities. | 94.0 | 95.0 |
| 14 | I think it is better not to rehabilitation when conditions (sleep, appetite, etc.) is not good. (reversed item) | 51.8 | 43.8 |
| 15 | I want to try several different exercises/practices. | 94.0 | 97.5 |
| 16 | I want to undergo the rehabilitation, even if I feel some pain and/or numbness. | 81.9 | 83.8 |
| 17 | I want to train by myself in addition to usual supervised training. | 94.0 | 95.0 |
| 18 | I think I must actively participate in rehabilitation. | 95.2 | 95.0 |
| 19 | I think rehabilitation is essential for recovering from diseases and disabilities. | 94.0 | 95.0 |
