# Supplementary material for: Development and validation of new evaluation scale for measuring stroke patients’ motivation for rehabilitation in rehabilitation wards
Source: PLoS One. 2022 Mar 17;17(3):e0265214. doi: 10.1371/journal.pone.0265214 (PMC8929594; doi:10.1371/journal.pone.0265214)
Supplement: S1 Table — The items of original Japanese version of MORE scale. (DOCX) [file pone.0265214.s002.docx]

**S1 Table. The original Japanese version of the MORE scale.**

| Item |  |
| --- | --- |
| 1 | 退院後の目標に向けてリハビリテーションに取り組みたい． |
| 2 | 自分が納得するまで体を良くしてから退院したい． |
| 3 | 家庭や社会での役割に復帰するために訓練をしたい． |
| 4 | 自分自身の目標は自分の頑張り次第で達成できる． |
| 5 | リハビリテーションの担当療法士の指導に応えたい． |
| 6 | リハビリテーションで行ったことを日常生活で応用させたい． |
| 7 | 日々行っている訓練の目標は担当療法士と共有できていると感じる． |
| 8 | 日々の訓練内容の変化にやりがいを感じる． |
| 9 | 他の患者さんが頑張っている姿は自分自身の励みになる． |
| 10 | 家族や友人のためにもリハビリテーションを頑張りたい． |
| 11 | 自分自身の体（または動作）は日に日に良くなっている． |
| 12 | できなかった動作があると，それが出来るように訓練したい． |
| 13 | いろいろな課題・訓練に挑戦したい． |
| 14 | 多少の痺れや痛みがあってもリハビリテーションは行いたい． |
| 15 | 訓練時間以外にも自分なりに訓練の時間を作りたい． |
| 16 | 毎日の訓練は自ら取り組む必要があると思う． |
| 17 | 今回の病気や障害を改善させるためにはリハビリテーションが必要である． |
