# Supplementary material for: Development and validation of new evaluation scale for measuring stroke patients’ motivation for rehabilitation in rehabilitation wards
Source: PLoS One. 2022 Mar 17;17(3):e0265214. doi: 10.1371/journal.pone.0265214 (PMC8929594; doi:10.1371/journal.pone.0265214)
Supplement: Attachment — Submitted filename: Response_to_Reviewers_220215.docx [file pone.0265214.s005.docx]

**Reviewer Comments:**

**Comments from Reviewer #2:**

I have few recommendations for the quality improvement in abstract, as follows

"No valid and reliable tools~": I hope the authors tone down this sentence. ex) It lacks study about-

Results:

-There is no sentences about reliability, thus please add contents about reliability.

-The readers might have difficulty to find results relevant to convergent, discriminant and criterion validity. Thus please add more explanation in results for those validity.

Authors’ comments:

Thank you for your constructive suggestions. To tone down the wording of the objective, the first two sentences of the objective section in the Abstract have been removed, because they would be considered background, rather than statement of the objective. We have added a description of the reliability to the results section and modified the wording in the text to make it easier for readers to understand convergent, discriminant, and criterion validity. We have revised the abstract as follows.

P3, L27-

***Objective:*** This study aimed to develop the Motivation in stroke patients for rehabilitation scale (MORE scale), following the Consensus-based standards for the selection of health measurement instruments (COSMIN).

P3, L29-

***Method:*** Study participants included rehabilitation professionals working at the convalescent rehabilitation hospital and stroke patients admitted to the hospital.

P3, L38-

***Results:*** Using the Delphi method, 17 items were incorporated into the MORE scale. According to EFA and CFA, a one-factor model was suggested. All MORE scale items demonstrated satisfactory item response, with item slopes ranging from 0.811 to 2.142, and item difficulty parameters ranging from -3.203 to 0.522. Cronbach’s alpha was 0.948. Regarding test-retest reliability, a moderate correlation was found between scores at the beginning and one month after hospitalization (rho = 0.612. p < 0.001). The MORE scale showed significant correlation with AS (rho = -0.536, p < 0.001), SDS (rho = -0.347, p < 0.001), and VAS (rho = 0.536, p < 0.001), confirming the convergent, discriminant, and criterion validity, respectively.
